# Factors and symptoms associated with work stress and health-promoting lifestyles among hospital staff: a pilot study in Taiwan

**DOI:** 10.1186/1472-6963-12-199

**Published:** 2012-07-16

**Authors:** Yueh-Chi Tsai, Chieh-Hsing Liu

**Affiliations:** 1 Department of Family Medicine, Mackay Memorial Hospital, Taipei City, Taiwan, 10449, Republic of China; 2 Department of Health Promotion and Health Education, National Taiwan Normal University, No. 162, Ho-Ping E. Road, Sec 1, Taipei, Taiwan, 106, Republic of China

**Keywords:** Healthcare workers, Health-Promoting Lifestyle Profile (HPLSP), Job Content Questionnaire (JCQ), Occupational stress

## Abstract

**Background:**

Healthcare workers including physicians, nurses, medical technicians and administrative staff experience high levels of occupational stress as a result of heavy workloads, extended working hours and time-related pressure. The aims of this study were to investigate factors associated with work stress among hospital staff members and to evaluate their health-promoting lifestyle behaviors.

**Methods:**

We conducted a cross-sectional study from May 1, 2010 to July 30, 2010 and recruited 775 professional staff from two regional hospitals in Taiwan using purposive sampling. Demographic data and self-reported symptoms related to work-related stress were collected. Each subject completed the Chinese versions of the Job Content Questionnaire (C-JCQ) and The Health-Promoting Lifestyle Profile (HPLSP). Linear and binary regression analyses were applied to identify associations between these two measurements and subjects’ characteristics, and associations between the two measurements and stress symptoms.

**Results:**

Self-reported symptoms of work-related stress included 64.4% of subjects reporting nervousness, 33.7% nightmares, 44.1% irritability, 40.8% headaches, 35.0% insomnia, and 41.4% gastrointestinal upset. C-JCQ scores for psychological demands of the job and discretion to utilize skills had a positive correlation with stress-related symptoms; however, the C-JCQ scores for decision-making authority and social support correlated negatively with stress-related symptoms except for nightmares and irritability. All items on the HPLSP correlated negatively with stress-related symptoms except for irritability, indicating an association between subjects’ symptoms and a poor quality of health-promoting lifestyle behaviors.

**Conclusions:**

We found that high demands, little decision-making authority, and low levels of social support were associated with the development of stress-related symptoms. The results also suggested that better performance on or a higher frequency of health-promoting life-style behaviors might reduce the chances of hospital staff developing stress-related symptoms. Our report may contribute to the development of educational programs designed to encourage members of high stress groups among the hospital staff to increase their health-promoting behaviors.

## Background

Healthcare workers in hospitals are exposed to high levels of occupational stress resulting from heavy workloads, extended working hours and high levels of time pressure. Hospital staff members, including physicians and nurses, are at a higher risk of suffering from depressive disorders than is the general population [1]. The hazards associated with the prolonged hours worked by resident physicians and interns have been documented. Depressed residents made 6.2 times as many medication errors per resident month as did residents who were not depressed [2]. Hospital staff nurses who had frequent overtime had difficulties in staying awake on duty and reduced sleep times, and had nearly triple the risk of making an error [3].

Recently, considerable concern about job stress has given rise to a theoretical approach that focuses on a demand-control-support model of job strain, as proposed by Karasek et al. This model predicts that job strain will occur when psychological work demands are high and the worker’s job control is low, while a low level of workplace social support will increase the risk of negative health outcomes. The psychological demand dimension relates to "how hard workers work" (mental work load), organizational constraints on task completion, and conflicting demands. Job control, discretion in utilizing ones’ skills, and decision-making authority are measured by a set of questions that assess the level of skill and creativity required on the job and the flexibility permitted the worker in deciding what skills to employ. [4]. A Job Content Questionnaire (JCQ) was developed by Karasek et al. based on the demand-control-support model. The model predicts that job strain will occur when the psychological demands of the job are high and the worker’s decision-making latitude is low, while a low level of support increases the risk of negative outcomes [4].

A study of nurses in Taiwan, found that occupational stress was associated with young age, marital status (widowed/divorced/separated), high psychological demand, low workplace support, and threat of assault at work. A lower score for general health was associated with low job control, high psychological demand, and perceived occupational stress. A lower mental health score was associated with low job control, high psychological demand, low workplace support, and perceived occupational stress [5].

While the JCQ is able to evaluate psychosocial aspects of workplace stress, it does not consider individual personalities or lifestyle factors that may influence responses to those stressors. Job stress has been linked to a range of adverse physical and mental health outcomes, such as cardiovascular disease, insomnia, depression, and anxiety [6]. Increasing employee participation and control through workplace reorganization based on the "demand-control-support" model improved both psychological and physical health [7].

Health-promoting behaviors were described by Walker et al. as behaviors that were directed toward sustaining or increasing the individual’s level of well-being, self-actualization and personal fulfillment [8]. A Health-Promoting Lifestyle Profile (HPLSP) scale was developed by Walker based on this concept [8]. Pender suggested that health-protecting (preventive) and health-promoting behaviors might be viewed as complementary components of a healthy life-style and proposed the Health Promotion Model as a paradigm for explaining health promoting behavior. Health-protecting behavior, an expression of the stabilizing tendency of humans, is directed toward decreasing the individual’s probability of encountering illness [9]. A study conducted by Lee et al. found that nurses in Taiwan had a high level of work pressure but they had better strategies for coping with stress as well. On the HPLSP, self-actualization and health responsibility correlated negatively with work stresses. [10].

The present study proposed that, for the professional staff in a hospital, the extent of job stress (measured by the JCQ) and performance in health-promoting lifestyle (measured by HPLSP) may correlate with stress-related symptoms. To the best of our knowledge, no previous study has examined the correlations among the factors related to stress in a cross section of hospital staff professionals.

## Methods

### Participants

In this cross-sectional study, a total of 1069 subjects who worked in regional hospitals in Chia-Yi and Hsin-Chu in Taiwan, were selected based on purposively sampling (deliberate, non-random sampling of the target population) from May 1^st^, 2010 to July 30^th^, 2010. The retrieval rate was 72.5% (775/1069) and 467 subjects were in Chia-Yi and 308 subjects in Hsin-Chu. After the study was explained to participants, all subjects provided written informed consent. The institutional review boards of the two hospitals approved the study protocol.

### Measurements

The participants were asked to complete a self-reported questionnaire about basic characteristics including job, marriage, education, location of hospital, length of work experience, average number of hours worked per day and symptoms related to work-related stress. These symptoms were chosen based on related articles in the literature [11-13] and discussion by a panel of experts. Cronbach’s α coefficient for this part of the questionnaire was 0.87. Participants were also asked to complete two questionnaires: the Chinese version of Karasek’s Job Content Questionnaire (C-JCQ) [14] and the HPLSP [15].

### Instruments

The C-JCQ [14] is a modification of the scale originally developed by Karasek et al. [4]. It consists of three dimensions: psychosocial work demands (5 items), job control (skill discretion, decision-making authority; 9 items), and workplace social support (coworker social support, supervisor social support; 8 items). Each item is measured on a four-point Likert scale (1: strongly disagree to 4: strongly agree). Cronbach’s α coefficient for the work demands subscale was 0.71, while those for the job control and workplace social support subscales were 0.69 and 0.81, respectively. Overall Cronbach's α was 0.72, and only those items with a Content Validity Index (CVI) over 0.8 were included in the final version of the C-JCQ.

A short form of the Chinese version of the HPLSP scale was developed by Wei & Lu in 2005 [15] as a modification of the HPLP scale originally designed by Walker et al. [8]. The scale consists of a set of 24 items that assess six dimensions of healthy behavior: Self-actualization, Health responsibility, Nutrition, Exercise, Interpersonal support, and Stress management. The Chinese version of the scale uses a four-point self-reported Likert scale scored as “never” (1), “sometimes” (2), “usually” (3), or “always” (4) to determine the frequency of reported behaviors. Internal reliability for the total scale was previously determined to be 0.90 with a range of 0.63 to 0.79 for the subscales [15].

### Statistical analysis

Subjects’ characteristics were summarized as mean ± standard deviation (SD) for continuous variables and count (n) with percentage (%) for categorical variables. Measurements on the JCQ and HPLSP were represented as mean ± SD and range (min. - max.) Spearman’s correlation analysis, point biserial correlation analysis, and point multiserial correlation analysis were utilized to show the coefficients of correlation. Binary logistic regression analysis was also utilized to identify symptoms of work-related stress while considering subjects’ characteristics, JCQ, and HPLSP. All statistical assessments were considered significant at *p* < 0.05. Statistical analyses were performed using SPSS 15.0 statistical software (SPSS Inc, Chicago, IL, USA).

## Results

Among the 775 subjects, 107 were male and 668 were female. Table 1 shows the demographic characteristics and stress-related symptoms of the 775 subjects. Overall, 376 (48.5%) worked as nursing staff, 248 (32%) as administrative staff, 116 (15%) as medical technicians, and 35 (4.5%) as physicians. Among all subjects, 400 (51.6%) were married and 360 (46.5%) were single; 669 (86.3%) had completed college or university. The average work experience of all subjects was 9.88 years (SD = 6.46) and the time period of average daily work was 8.77 hours (SD = 1.66). For self-reported symptoms of work-related stress, 499 (64.4%) of the subjects reported being nervous, 261 (33.7%) had nightmares, 342 (44.1%) had irritability, 316 (40.8%) had headache, 271 (35.0%) had insomnia, and 342 (41.4%) had gastrointestinal upset.

**Table 1:** Subjects’ demographic characteristics and work-related symptoms (N = 775)

**Variables**	**(N = 775)**
Job position	
Physician	35 (4.5)
Nurse	376 (48.5)
Medical technician staff	116 (15.0)
Administrative staff	248 (32.0)
Marriage status	
Married	400 (51.6)
Not married	360 (46.5)
Other	15 (1.9)
Education	
High school	59 (7.6)
College or University	669 (86.3)
Master’s or PhD	47 (6.1)
Location of hospital	
Chai-Yi	467 (60.3)
Hsin-Chu	308 (39.7)
Work experience, years	9.88 ± 6.46
Daily work time, hours	8.77 ± 1.66
Symptoms of work-related stress	
Nervousness	499 (64.4)
Nightmares	261 (33.7)
Irritability	342 (44.1)
Headaches	316 (40.8)
Insomnia	271 (35.0)
Gastrointestinal upset	321 (41.4)

Data are represented as n (%) for categorical variables and mean ± SD for continuous variables.

Table 2 summarizes the measurements from the C-JCQ and HPLSP questionnaires. Average scores for each sub-category of the C-JCQ were: 13.87 ± 2.11 for psychological demands on the job, 16.82 ± 2.37 for discretion in use of skills, 7.83 ± 1.36 for decision-making authority, 12.17 ± 1.62 for coworker social support, and 11.26 ± 2.09 for supervisor social support. The outcomes on HPLSP measurement showed that both exercise (7.83 ± 2.15) and stress management (9.90 ± 2.11) had relatively low scores. The total score for health-promoting life-style reached only 64.47 ± 11.19 with a range from 28 to 106.

**Table 2:** **Summary of measurements on Job Content Questionnaires (JCQ) and health-promoting lifestyle profile (HPLSP) for all 755 subjects**^**a**^

**Variables**	**mean ± SD**	**Range**
Job Content Questionnaire		
Psychological demands of the job	13.87 ± 2.11	(7–20)
Discretion in use of skills	16.82 ± 2.37	(6–23)
Decision-making authority	7.83 ± 1.36	(3–12)
Coworker social support^†^	12.17 ± 1.62	(4–16)
Supervisor social support^†^	11.26 ± 2.09	(4–16)
Health-promoting lifestyle profiles		
Self-actualization	10.59 ± 2.51	(4–16)
Health responsibility	10.66 ± 2.70	(5–20)
Exercise	7.83 ± 2.15	(4–15)
Nutrition	14.44 ± 3.54	(6–24)
Interpersonal support	11.05 ± 2.07	(4–16)
Stress management	9.90 ± 2.11	(4–16)
Total score of health-promoting lifestyle profile	64.47 ± 11.19	(28–106)

^a^ Data are represented as mean ± SD and range (min. - max.).

^†^ Those two items asked for the need for social support. In other words, a higher score stands for not enough social support from either coworkers or supervisor.

Table 3 shows the correlations between subjects’ characteristics and C-JCQ categories. The results show that gender was positively correlated with psychological demands, but negatively correlated with discretion in use of skills as female staff had greater psychological demands but less discretion in their use of skills. Job position ranked in the order of physicians, nurses, medical technician staff and administrative staff was negatively correlated with psychological demands, discretion in use of skills and decision-making authority, as physicians had greater psychological demands, discretion in their use of skills and decision-making authority than did nurses, followed by medical technician staff, and administrative staff.

**Table 3:** Correlation of Job Content Questionnaire with subjects’ characteristics (N = 755)

	**Psychological Demands of Job**		**Discretion In Use of Skills**		**Decision-making authority**		**Coworker social support**^**a**^		**Supervisor social support**^**a**^	
	**r**	**p-value**	**r**	**p-value**	**r**	**p-value**	**r**	**p-value**	**r**	**p-value**
Gender	.085	0.018^*^	-.127	<.001^***^	-.057	0.113	.011	0.764	-.015	0.682
Job position	-.235	<.001^***^	-.297	<.001^***^	-.103	0.004^**^	.014	0.702	-.010	0.785
Work experience, years	.002	0.947	.146	<.001^***^	.115	0.001^**^	-.095	0.008^**^	-.079	0.027^*^
Daily work times, hours	.326	<.001^***^	.296	<.001^***^	.107	0.003^**^	-.016	0.647	-.033	0.358

r, coefficient of correlation of Job Content Questionnaire with gender was derived through the point biserial correlation analysis (male, 1; female, 2); with job position through the point multiserial correlation analysis (physician, 1; nurse, 2; medical technician staff, 3; administrative staff, 4); with work experience or with daily work times through Spearman’s correlation analysis.

^*^P < 0.05, ^**^P < 0.01^, ***^P < 0.001, indicate significant correlation between health-promoting life-style profiles and characteristics.

^a^These two items indicate perceived needs for social support. In other words, a higher score stands for not enough social support from either coworkers or supervisors.

Longer work experience was positively correlated with discretion in use of skills and decision-making authority but showed a negative correlation with coworker social support and supervisor social support. (Table 3) Daily work times were positively correlated with psychological demands, discretion in use of skills and decision-making authority. (Table 3)

The correlation of subjects’ characteristics with HPLSP categories is shown in Table 4. The total HPLSP score was positively correlated with job position (r = 0.168, P < 0.001) but negatively correlated with daily work time (r=−0.127, P < 0.001). Physicians had the lowest score in HPLSP among the four positions, followed by nurses, medical technician staff, and administrative staff in that order.

**Table 4:** Correlation of Health-promoting life-style profiles with subjects’ characteristics (N = 755)

**Characteristics**	**Self-actualization**		**Health responsibility**		**Exercise**		**Nutrition**		**Interpersonal support**		**Stress management**		**Total score for health-promoting life-style**	
	**r**	**P-value**	**r**	**P-value**	**r**	**P-value**	r	**P-value**	**r**	**P-value**	**r**	**P-value**	**r**	**P-value**
Gender	−0.057	0.111	0.009	0.805	−0.059	0.100	−0.066	0.068	0.051	0.156	0.005	0.889	−0.032	0.368
Job position	0.122	0.001^**^	0.019	0.599	0.084	0.019^*^	0.269	<.001^***^	0.065	0.071	0.121	0.001^**^	0.168	<.001^***^
Work experience, years	0.062	0.087	0.078	0.030^*^	0.015	0.672	0.198	<.001^***^	−0.018	0.613	−0.001	0.977	0.087	0.015
Daily work times, hours	−0.072	0.045^*^	−0.038	0.290	−0.094	0.009^**^	−0.188	<.001^***^	−0.018	0.623	−0.095	0.008^**^	−0.127	<.001^***^

r, coefficient of correlation of health-promoting life-style profiles with gender was derived through the point biserial correlation analysis (male, 1; female, 2); with job position through the point multiserial correlation analysis (physician, 1; nurse, 2; medical technician staff, 3; administrative staff, 4); with work experience or with daily work times through Spearman’s correlation analysis.

^*^P < 0.05, ^**^P < 0.01^, ***^P < 0.001, indicate significant correlation between health-promoting life-style profiles and characteristics.

Among the subcategories of HPLSP, most (Self-actualization, Exercise, Nutrition, Stress management) were positively correlated with job position. Physicians had less self-actualization, fewer chances to exercise, cared less about nutrition, and did not manage stress well. They were followed by nurses, medical technician staff, and administrative staff in that order. (Table 4) Health responsibility and interpersonal support were positively correlated with work experience, as staff with longer work experience took greater health responsibility and had more interpersonal support. Most subcategories (Self-actualization, Exercise, Nutrition, Stress management) were negatively correlated with daily work times, as staff who worked longer had less self-actualization, did less exercise, cared less about nutrition, and did not manage stress well. (Table 4)

Table 5 shows the association between subjects’ characteristics (gender, job, work experience, daily work time), C-JCQ and HPLSP scores, and stress symptoms as determined by binary logistic regression analysis. Female workers had more stress-related symptoms than did male workers, except for gastrointestinal upset. Participants with longer work experience had a significantly higher chance of having stress-related symptoms, except for irritability and gastrointestinal upset. Participants with longer daily work times had more stress-related symptoms, except for irritability and insomnia.

**Table 5:** **Association of subjects’ characteristics, Job Content Questionnaire, and Health-promoting life-style profiles with symptoms of work-related stress through binary logistic regression analysis**^**a**^

**Variables**	**Nervousness**	**Nightmares**	**Irritability**	**Headaches**	**Insomnia**	**Gastrointestinal upset**
	**OR (95% CI.)**	**OR (95% CI.)**	**OR (95% CI.)**	**OR (95% CI.)**	**OR (95% CI.)**	**OR (95% CI.)**
Gender						
Male	1	1	1	1	1	1
Female	2.56 (1.69 – 2.56)*	1.69 (1.05 – 1.69)*	1.92 (1.25 – 1.92)*	1.82 (1.16 – 2.86)*	1.82 (1.12 – 2.86)*	1.33 (0.87 – 2.04)
Job						
Physician	1	1	1	1	1	1
Nurse	2.79 (1.38 - 5.64)*	3.95 (1.60 - 9.73)*	2.45 (1.19 - 5.07)*	4.64 (1.98 - 10.89)*	5.51 (2.09 - 14.51)*	1.50 (0.75 - 3.02)
Medical technician staff	1.34 (0.63 - 2.86)	1.76 (0.67 - 4.65)	1.13 (0.51 - 2.50)	1.80 (0.72 - 4.50)	2.29 (0.82 - 6.41)	0.68 (0.31 - 1.46)
Administrative staff	1.08 (0.53 - 2.18)	1.38 (0.54 - 3.49)	0.85 (0.40 - 1.79)	1.61 (0.67 - 3.84)	1.67 (0.62 - 4.51)	0.50 (0.24 - 1.04)
Work experience, years	0.96 (0.94 - 0.99)*	0.98 (0.95 - 1.00)*	0.97 (0.95 - 0.99)	0.97 (0.95 - 1.00)*	0.97 (0.95 - 1.00)*	0.99 (0.96 - 1.01)
Daily work time, hours	1.09 (0.99 - 1.19)*	1.09 (0.99 - 1.19)*	1.06 (0.97 - 1.15)	1.12 (1.02 - 1.23)*	1.07 (0.98 - 1.17)	1.15 (1.05 - 1.27)*
Job Content Questionnaire						
Psychological demands of job	1.50 (1.37 - 1.63)*	1.39 (1.28 - 1.51)*	1.52 (1.40 - 1.66)	1.49 (1.37 - 1.62)*	1.37 (1.27 - 1.48)*	1.55 (1.42 - 1.69)*
Discretion in using skills	1.10 (1.04 - 1.17)*	1.13 (1.06 - 1.21)*	1.04 (0.98 - 1.11)	1.10 (1.03 - 1.17)*	1.07 (1.00 - 1.14)*	1.13 (1.06 - 1.21)*
Decision-making authority	0.86 (0.77 - 0.97)*	0.79 (0.71 - 0.89)*	0.76 (0.68 - 0.85)	0.81 (0.72 - 0.90)*	0.83 (0.74 - 0.93)*	0.86 (0.78 - 0.96)*
Coworker social support^†^	0.99 (0.91 - 1.09)*	1.01 (0.92 - 1.11)	0.89 (0.81 - 0.97)	0.89 (0.81 - 0.97)*	0.95 (0.87 - 1.04)*	0.93 (0.85 - 1.02)
Supervisor social support^†^	0.98 (0.92 - 1.06)	0.96 (0.89 - 1.03)	0.83 (0.77 - 0.89)	0.91 (0.85 - 0.98)*	0.92 (0.86 - 0.99)*	0.86 (0.80 - 0.92)*
Health-promoting life-style profiles						
Self-actualization	0.83 (0.78 - 0.88)*	0.83 (0.78 - 0.88)*	0.78 (0.73 - 0.83)	0.81 (0.76 - 0.86)*	0.79 (0.74 - 0.84)*	0.85 (0.80 - 0.90)*
Health responsibility	0.94 (0.89 - 0.99)*	0.94 (0.88 - 0.99)*	0.91 (0.86 - 0.96)	0.92 (0.87 - 0.97)*	0.94 (0.89 - 0.99)*	0.98 (0.93 - 1.03)
Exercise	0.89 (0.83 - 0.96)*	0.91 (0.85 - 0.97)*	0.90 (0.84 - 0.96)	0.92 (0.86 - 0.98)*	0.94 (0.88 - 1.01)*	0.93 (0.87 - 0.99)*
Nutrition	0.88 (0.84 - 0.92)*	0.89 (0.85 - 0.93)*	0.86 (0.82 - 0.89)	0.86 (0.82 - 0.90)*	0.85 (0.81 - 0.89)*	0.89 (0.85 - 0.93)*
Interpersonal support	0.92 (0.86 - 0.99)*	0.86 (0.80 - 0.93)*	0.88 (0.82 - 0.95)	0.88 (0.82 - 0.95)*	0.92 (0.85 - 0.98)*	0.98 (0.91 - 1.04)
Stress management	0.85 (0.79 - 0.92)*	0.82 (0.76 - 0.89)*	0.77 (0.71 - 0.83)	0.77 (0.71 - 0.83)*	0.77 (0.71 - 0.83)*	0.82 (0.77 - 0.89)*
Total score of health-promoting life-style	0.96 (0.95 - 0.98)*	0.96 (0.95 - 0.97)*	0.95 (0.94 - 0.96)	0.95 (0.94 - 0.97)*	0.95 (0.94 - 0.97)*	0.97 (0.96 - 0.98)*

^a^ Results are represented as estimated odds ratio (OR) with 95% confidence interval (95% CI.) on binary logistic regression analysis.

* *P* < 0.05 for estimated OR.

^†^These two items indicate needs for social support. In other words, a higher score stands for not enough social support from either coworkers or supervisors.

For C-JCQ items, scores on psychological demands of the job and decisions to utilize skills had a positive correlation with stress-related symptoms, except for irritability; however, decision-making authority scores had a negative correlation with stress-related symptoms, except for irritability. Both the scores for coworker social support and supervisor social support had negative correlations with stress-related symptoms except for nightmares and irritability. All items on the health-promoting life-style profiles had a negative correlation with stress-related symptoms, except for irritability.

## Discussion

High job demands, little decision-making authority, and low social support were associated with the development of stress-related symptoms. Relationships were also shown between job category and dimensions of the JCQ. Male workers had fewer psychological demands on the job than did females but had greater discretion in utilizing their skills. Hospital nursing staff, medical technicians and administrative staff had significantly less discretion in utilizing their skills and decision-making authority than did physicians. Longer work experience was associated with significantly higher discretion in utilizing skills and decision-making authority among all workers. Longer daily work times were associated with significantly higher psychological demands on the job, discretion in utilizing skills, and decision-making authority. Psychological demands on the job were somewhat associated with gender and daily work time. In terms of symptoms, being female, having a longer work experience, and working longer hours each day were associated with significantly greater stress-related symptoms, except for irritability. Nervousness, headache, and, to a lesser extent, gastrointestinal upset were reported more frequently.

Staff responses to HPLSP categories revealed that nurses performed less well in self-actualization and nutrition categories when compared to other types of workers. Participants with longer work experience performed better in self-actualization, health responsibility and nutrition categories. Staff with longer daily work times performed less well in nutrition, indicating that time constraints on the job interfered with their ability to eat well. The total scores for health-promoting lifestyle indicated that hospital staff did have an interest in health-promoting measures but did not always perform them well. All items on the HPLSP correlated negatively with stress-related symptoms except for irritability, indicating an association between subjects’ symptoms and their self-reported, low-quality, health-promoting lifestyle behaviors.

As in the study by Shen et al. [5], lower general health scores measured by the JCQ were associated with low job control, high psychological demand, and perceived occupational stress. A lower mental health score was associated with low job control, high psychological demand, low workplace support, and perceived occupational stress. In the present study, low job control was represented by low scores for decision-making authority and discretion in utilization of skills. We found that scores for psychological demands on the job and discretion in utilizing skills correlated positively with stress-related symptoms while decision-making authority scores correlated negatively with stress-related symptoms.

McElligott et al. [16] examined the health-promoting lifestyle behaviors of acute-care nurses using the Health Promotion Model. Their results showed overall low scores for health-promoting behavior, with particular weaknesses in stress management and physical activity. In our study, we also found that almost all items on the HPLSP correlated negatively with stress-related symptoms, indicating an association between high-quality health-promoting lifestyle behavior and fewer stress-related symptoms.

In the present study, nurses sensed a lack of social support from peers and supervisors and, in expressing a need for more social support, placed a high value on relationships at work as being an important aspect of the work environment. Work relationships were also cited as a direct source of stress by Hope et al. as hospital nurses who experienced high work stress were more apt to seek professional support and the support of family and friends or “having a good cry” [17]. Seeking support from coworkers or supervisors may actually represent health-protecting behavior that could help diffuse the impact of stressors in the workplace. Based on our results regarding the expressed need for support among hospital staff, measures such as conflict resolution and peer support groups could help increase health-promoting skills and thereby reduce the development of stress-related symptoms.

Solutions must fit the problem and different settings have produced different explanations for work-related stress. A recent study by Chen et al. explored job stress and specific stressors along with coping strategies and overall job satisfaction among nurses and found that the main stressors were related to the type of hospital, patient safety issues, and administrative feedback. They recommended implementation of standard operating procedures, security measures and increases in the quantity and quality of stress relief courses [18]. Other studies have suggested that health-promotion skills should be integrated into nursing education. This could have a ripple effect that may improve both the nurses’ own health status and enhance their role as health promotion advocates [17,19].

Many researchers in Taiwan have studied job stress, coping strategies and health promoting behavior among hospital staffs [5,14,18,20,21], and in other occupations [22]. The present study is the first to comprehensively investigate associations between scores on the C-JCQ and HPLSP and stress-related symptoms among hospital staff members in Taiwan.

There are several limitations to our study. First, 48.5% of the subjects worked as nursing staff and only 4.5% as physicians; therefore, nursing staff responses may disproportionally affect the overall scores on the questionnaires, and, to a lesser extent, reflect the job content and life-style profile of male physicians. Second, our study population of 775 healthcare workers was recruited from only two regional hospitals, and this may preclude generalization of the results to larger and smaller institutions such as medical centers, local hospitals, and clinics. Third, odds ratios in logistic regression may not be appropriate for a cross-sectional study, and a prospective study should be conducted in the future. Finally, a future study will be needed to demonstrate whether a high quality of health-promoting lifestyle can really reduce the stress-related symptoms associated with high demand, low control and low social support.

## Conclusions

Little decision-making authority and a lack of social support from either coworkers or supervisors are associated with the development of stress-related symptoms. Better performance in or higher frequency of health-promoting life-style behaviors might reduce the chances of developing stress-related symptoms. We suggest that our results may be useful in the development of educational programs designed to encourage highly stressed hospital staff members to pay more attention to health-promoting lifestyles and to increase health-promoting behaviors as protection against the demands of the hospital work environment.

## Competing interests

The authors declare that they have no competing interests.

## Authors’ contributions

YC Tsai designed the study, wrote the protocol, managed the literature searches, data acquisition and analysis, and wrote the manuscript. Dr. CH Liu was the supervisor for the project, was closely involved in creating the hypothesis and study design, and undertook the manuscript editing and review of the rough draft. All authors read and approved the final manuscript.

## Pre-publication history

The pre-publication history for this paper can be accessed here:

http://www.biomedcentral.com/1472-6963/12/199/prepub
